# Supplementary material for: Smart Bio-Nanocoatings with Simple Post-Synthesis Reversible Adjustment
Source: Biomimetics (Basel). 2025 Mar 7;10(3):163. doi: 10.3390/biomimetics10030163 (PMC11940101; doi:10.3390/biomimetics10030163)
Supplement: Supplementary file 1 [file biomimetics-10-00163-s001.zip › biomimetics-3499059-supplementary.pdf]

biomimetics

MDPI

Supplementary Materials:

Supplementary Table S1. Mass-spectrometry analysis of *T. castaneum* corneal proteins

Identified genes	MW (kDa)	Percentage of total spectra (%)	Percent coverage (%)	Total unique peptide count
TC003109 (CP7)	15	0.74	61	8
Yellow-e	45	0.45	40	13
TC001574	17	0.27	69	9
Tubulin	50	0.27	26	8
TC001251	17	0.25	60	7
TC030667	20	0.27	37	6
GLEAN 07135	20	0.22	60	9
TC005525	18	0.22	29	6
TC012647	21	0.27	60	9
Calponin	20	0.18	50	7
